# Modelling the overlap and divergence of autistic and schizotypal traits on hippocampal subfield volumes and regional cerebral blood flow

**DOI:** 10.1038/s41380-023-02302-w

**Published:** 2023-10-27

**Authors:** Igor Nenadić, Tina Meller, Ulrika Evermann, Julia-Katharina Pfarr, Andrea Federspiel, Sebastian Walther, Sarah Grezellschak, Ahmad Abu-Akel

**Affiliations:** 1 Cognitive Neuropsychiatry Lab, Department of Psychiatry and Psychotherapy, Philipps Universität Marburg, Marburg, Germany; 2 Center for Mind, Brain, and Behavior (CMBB), University of Marburg and Justus Liebig University Giessen, Marburg, Germany; 3 Marburg University Hospital – UKGM, Marburg, Germany; 4 Translational Research Center, University Hospital of Psychiatry and Psychotherapy, University of Bern, Bern, Switzerland; 5 Institute of Diagnostic and Interventional Neuroradiology, Inselspital, Bern University Hospital, and University of Bern, Bern, Switzerland; 6 School of Psychological Sciences, University of Haifa, Mount Carmel, Haifa Israel; 7 The Haifa Brain and Behavior Hub, University of Haifa, Mount Carmel, Haifa Israel

**Keywords:** Neuroscience, Diagnostic markers

## Abstract

Psychiatric disorders show high co-morbidity, including co-morbid expressions of subclinical psychopathology across multiple disease spectra. Given the limitations of classical case-control designs in elucidating this overlap, new approaches are needed to identify biological underpinnings of spectra and their interaction. We assessed autistic-like traits (using the Autism Quotient, AQ) and schizotypy - as models of subclinical expressions of disease phenotypes and examined their association with volumes and regional cerebral blood flow (rCBF) of anterior, mid- and posterior hippocampus segments from  structural MRI scans in 318 and arterial spin labelling (ASL) in 346 nonclinical subjects, which overlapped with the structural imaging sample (*N* = 298). We demonstrate significant interactive effects of positive schizotypy and AQ social skills as well as of positive schizotypy and AQ imagination on hippocampal subfield volume variation. Moreover, we show that AQ attention switching modulated hippocampal head rCBF, while positive schizotypy by AQ attention to detail interactions modulated hippocampal tail rCBF. In addition, we show significant correlation of hippocampal volume and rCBF in both region-of-interest and voxel-wise analyses, which were robust after removal of variance related to schizotypy and autistic traits. These findings provide empirical evidence for both the modulation of hippocampal subfield structure and function through subclinical traits, and in particular how only the interaction of phenotype facets leads to significant reductions or variations in these parameters. This makes a case for considering the synergistic impact of different (subclinical) disease spectra on transdiagnostic biological parameters in psychiatry.

## Introduction

The overlap of symptoms and phenotypes between mental disorders and the problem of co-morbidity have become paramount for an understanding of their biological basis [1–3] as well as precision psychiatry interventions [4]. In clinical practice, this challenge spans the entire range of diagnostic assessments, prognostic evaluation, and implementation of personalised treatments, which consider individual specifics of each patient. Similarly, neurobiological research on mental disorders faces an increasing demand to conceptualise and understand comorbidities of mental disorders.

While conventional case-control-studies have grown to include large cohorts in mega-analyses, for example in genomics and brain imaging [5, 6], it has become increasingly clear, that these designs have inherent shortcomings when addressing the problem of comorbidity. In many of the above studies, comorbidity might often be treated as a co-variate (or neglected), failing to take into account interacting effects between disease spectra, which might manifest in additive or diametrically opposed effects on a biological parameter. Examples of this include the schizophrenia/psychosis spectrum and autisms spectrum disorders [7], or the overlap between affective disorders and anxiety disorders [8], where a main diagnosis is frequently accompanied by clinical or subclinical manifestation of a second phenotype. Addressing the problem of comorbidities is, however, not just a matter of adjusting the variability or heterogeneity in case-control studies. Research over recent years has made it increasingly clear that many of the established typical comorbidities are not just a result of chance, but rather reflect an inherent genetic or brain-level predisposition spanning conventional diagnostic categories [7]. On the genomic level, recent GWAS (genome wide association studies) have shown overlaps of common genetic risk variance between psychotic, affective, anxiety, and substance related disorders [6, 9]. In parallel, neuroimaging research has shown benefit from transdiagnostic studies demonstrating overlaps versus more specific structural or functional patterns of abnormalities characterising these conditions [10–12].

In order to conceptualise comorbidities (in particular those that occur clearly well above chance level), it might be important to consider single disease dimensions rather than diagnostic categories. In schizophrenia, for example, different brain correlates and mechanisms have been studied for positive vs. negative symptom dimensions [13–17]. This is also the case for subclinical risk phenotypes like schizotypy [18–20].

Dimensional approaches to psychopathology and underlying neurobiology, including Research Domain Criteria (RDoC) [21, 22] and other systems [23] have proposed transdiagnostic approaches, which in part also address the problem of comorbidity. However, their focus on transdiagnostic modelling, which has improved our understanding of neural underpinnings across or independent of clinical diagnostic boundaries, has not yet led to testable models of how certain phenotype or behavioural facets interact in generating variation in brain structure/function. In the case of the schizophrenia/psychosis versus the autism spectrum disorder (ASD) overlap, different models have been proposed to explain the co-occurrence as well as interactive effects on disease courses and treatment [7]. Previous research indicates that ASD and schizophrenia/psychosis exhibit both opposing and partially overlapping phenotypes, with ASD symptoms overlapping with negative symptoms and diametrically opposed to positive symptoms of schizophrenia/psychosis [24, 25]. This pattern has also been demonstrated in nonclinical cohorts, where the expression of subclinical phenotypes is studied and similar patterns have emerged [26, 27].

Most previous studies addressing problems related to neurobiological overlap versus distinct signatures across multiple mental disorders have suffered, however, from particular drawbacks related to commonly used case-control designs. For one, direct comparisons between particular disorders are hampered by disease-characteristic differences in age of onset, such as studies comparing adolescent-/adulthood-onset psychosis versus childhood-onset ASD [9, 28–31], or psychotic disorders versus adulthood-onset affective disorders [2]. Also, differences in disease course as well as treatment may limit the use of many available large-scale patient cohorts to specifically address problems related to comorbidity.

In the present study, we chose a novel approach for modelling co-occurrence of disease spectrum phenotypes. This approach uses well-established subclinical phenotypes that a) are multi-dimensional (i.e., characterise different aspects within a disease-related symptom spectrum), and b) are assessed in a nonclinical cohort with subclinical expression of disease-related phenotypes already established as having a biological link to the disease part of the spectrum. In this cohort, we then analysed both high-resolution structural brain scans and regional cerebral blood flow to illustrate underlying neurobiological patterns of multi-dimensional traits linked to disease and disease risk. Analysing subclinical expressions of disease phenotypes confers several advantages over mentioned case-control designs, such as eliminating the confounding effects of medication and illness chronicity, yet it requires specific deep phenotyping using established and validated trait (or state) markers of specific disease spectra or continua.

Based on extensive previous work relating subclinical autistic traits to genetic and brain structural markers linked to the clinical autism spectrum [32–34], as well as markers of psychosis proneness (measured with the positive scale of psychometrically established schizotypy questionnaire), which have been related to brain imaging markers [19, 35, 36], we test a computational modelling hypothesis integrating the concepts of overlapping versus diametrically opposed single symptom dimensions [7, 26] in the case of the hippocampus. Clearly, among the multiple brain regions associated with mental illness, the hippocampus has a key role – particularly in the psychosis spectrum. Prior work indicated that cerebral blood volume and cerebral blood flow are increased in subjects at risk for psychosis [37–40]. Furthermore, Schobel and colleagues demonstrated that increased cerebral blood volume indicates future grey matter loss in the hippocampus [39]. Increased cerebral blood flow/volume are also found in subjects with early psychosis [41, 42], thus rendering increased cerebral blood flow/volume an interesting marker of emerging and early psychosis. Most recently, a mouse model of ErbB4 mutants—a schizophrenia susceptibility gene—suggests that dysfunctional inhibitory interneurons drive increased cerebral blood flow and glutamine levels in the ventral hippocampus [43].

Moreover, accumulating evidence highlights the contribution of the hippocampus to social and cognitive deficits in ASD [44], which could be linked to aberrant decreases in hippocampal grey matter volume [45, 46] and/or cerebral blood flow [47, 48]. Finally, recent advances in MR-morphometry allow the separation into functionally distinct and previously characterised subregions [49, 50], which allow testing our hypotheses in the context of risk phenotypes like schizotypy, early psychosis, and schizophrenia [39, 41, 42, 51], and ASD [47] on volumes and perfusion in different parts of the hippocampus. Given evidence for the co-occurrence of autistic and positive schizotypal traits and that these dimensions might be anticorrelated within the same individual, we hypothesize that autistic and positive schizoptypal traits will be synergistically associated with hippocampal volume and CBF. This approach offers a more comprehensive understanding of the effect of individual combinations of disease spectrum phenotypes on hippocampal structural and functional variations.

## Methods

### Study cohort

We included a total of *N* = 318 (204 female/114 male; mean age = 23.95, SD = 3.85) psychiatrically healthy subjects within the age range of 18–40 years. Participants were recruited from the local community through advertisements and circular emails, and all were of central European descent. Using the German version of the Structured Clinical Interview for DSM-IV screening tool (SCID-I; [52, 53]), we ensured the absence of current or former psychiatric disorders; further exclusion criteria were: past and current substance abuse, history of traumatic brain injury and any neurological pathology, psychotropic medication, as well as other untreated medical conditions. The MWT-B [54], a German-language word-list based estimate of IQ, was used to ascertain an IQ > 80 as well as estimate IQ values for participants. All subjects gave written informed consent to a study protocol (according to the latest Declaration of Helsinki; [55]) approved by the local ethics committee of the School of Medicine, Philipps-University Marburg.

The ASL (arterial spin labelling) analysis was based on a sample of 346 psychiatrically healthy subjects, within the age range of 18–40 years (222 female/124 male; mean age = 23.94, SD = 3.89), which overlapped with the structural imaging sample (*n* = 298).

### Phenotyping

Schizotypy was measured using three subscales: the Schizotypal Personality Questionnaire – Brief version (SPQ-B; [56]), the Oxford-Liverpool Inventory of Feelings and Experiences (O-LIFE; [57, 58]), and the recently developed Multidimensional Schizotypy Scales (MSS; [59, 60]). This approach takes into account that currently used schizotypy inventories might show minor divergence, which might be based on the conceptualisation, for example the DSM criteria of schizotypal personality disorder (in the case of SPQ), a fully dimensional model of schizotypy (for O-LIFE), or a spectrum pathology model validated using classical test theory, item response theory, and differential item function approaches (MSS). Composite scores of positive, negative and disorganised schizotypy were calculated by averaging the standardised values of the measures’ respective subscales.

Autistic traits were assessed using the Autism Quotient Spectrum (AQ) [61], which consists of five subscales: Social skills, communication, attention switching, attention to detail and imagination. Based on recent psychometric recommendations [62], and evidence that different domains of autism are underpinned by different genetic influences [63], we used the subscales in the analyses to provide a more nuanced information of the specific domains that might be at play.

In addition, we applied Beck’ Depression Inventory (BDI) to assess concurrent (subclinical) depressive symptoms, for removal of effects in later analysis [64, 65].

### Magnetic resonance imaging (MRI) data acquisition and pre-processing

All images were acquired with a 3 T Siemens Tim Trio MRI scanner with 12-channel quadrature head coil, located at the BrainImaging Core Facility of the University of Marburg, Department of Psychiatry and Psychotherapy. For structural T1 images, a 3D MP-RAGE sequence with 176 sagittal slices was used to obtain voxels with an isometric size of 1 mm^3^. Acquisition time of this sequence was 4:26 min with TR = 1900ms, TE = 2.26 ms, TI = 900 ms, field-of-view= 256 mm × 256 mm, isotropic voxel size 1.0 mm × 1.0 mm × 1.0 mm, and flip angle = 9°. A total of 364 images underwent visual cheques for artefacts and homogeneity or anatomical abnormalities before they were passed on to the pre-processing pipeline included in Freesurfer version 6.0 (https://surfer.nmr.mgh.harvard.edu; [66, 67]). We used the main reconstruction (recon-all command) pipeline for tissue segmentation, and acquired bilateral volumes of the whole hippocampal formation, head, body and tail (HBT) subdivisions based on the automated probabilistic brain atlas [68] implemented in FreeSurfer 6.0.

The FreeSurfer segmentation algorithm is highly reliable, yet we aimed to optimise its accuracy [69] by adding an additional step for quality assurance. To this end, we compared raw and prescan-normalised (a scanner-based image homogeneity correction) versions of T1 images for each participant, excluding data of 46 individuals whose unnormalized-to-normalised image differences exceeded 3% for whole hippocampal volumes. The final sample comprised whole and HBT hippocampal volumes from 318 participants. Total Intracranial volume (ICV) was controlled for in all statistical analyses of a) whole and b) HBT hippocampal volumes.

Volumes of overall and the head, body and tail subdivisions of left and right hemisphere hippocampi were computed, as previously described [19].

Perfusion images were acquired with a pulsed arterial spin labelling (PASL) sequence at rest, using Siemens’ Proximal Inversion with Control of Off-Resonance Effects (PICORE Q2T) protocol. It included 16 slices with 7 mm thickness and a distance factor of 25%, with TR = 3000 ms, TE = 11 ms, inversion time 2 TI2 = 2200 ms, TI1 = 700 ms, and saturation stop time=1600ms, a field of view of 230 mm^2^ and flip angle of 90°, 153 measurements and a resulting voxel size of 3.6 mm × 3.6 mm × 6.0 mm. The first volume is an M0 image (i.e., equilibrium brain tissue magnetisation image) that was used for CBF quantification.

During pre-processing, all 153 images underwent motion correction and realignment, CBF quantification in native space (CBF quantification was performed using formula [1] as described in [70]), all 76 single CBF images including mean CBF images were co-registered with the individual anatomical T1 scans, normalised in MNI space and smoothed with a Gaussian kernel of 6mmx6mmx6mm.

Perfusion values of whole hippocampus and head/body/tail segments were extracted using MarsBar and right and left whole hippocampus masks provided by the Neuromorphometrics atlas (Neuromorphometrics Inc.), which were additionally split into three parts according to y-planes at coordinates y = −15 and y = −30, aiming to mirror Freesurfer’s partitions.

### Statistical analysis

We performed two separate MANCOVAs, using SPSS 26. The first examined the association of the standardised scores of positive schizotypy, the standardised AQ’s five subscales, and their 2-way interactions with overall volume variation of the left and right hippocampi, and their subfields (head, body and tail). The second MANCOVA included the same set of predictors as the first, but this time it examined their association with CBF variation in the left and right hippocampi, and their subfields (head, body and tail). Both MANCOVAs were performed while controlling for age, sex and total intracranial volume (ICV). IQ, depressive symptoms (BDI), negative, and disorganised schizotypy were not correlated with hippocampal volumes or CBF (ps > 0.05) and thus were not included in either of the MANCOVA models. Visualisation of the significant interactions from the MANCOVAs was performed with response surface analysis methodology (RSA) [71], using RStudio Version 2023.03.0 + 386. RSA enables us to map, in 3-D space, the response surface pattern of the 2-way interacting symptoms/dimensions with level of hippocampus volumes/CBF, as well as to test, based on the parameters of the interaction terms, whether the combined effect of the interacting terms is linear or curvilinear along two axes: the axis of balance or congruence, where the values of Predictor 1 = Predictor 2, and the axis of bias or incongruence, where the values of Predictor 1 = - Predictor 2. The distribution of raw values for the hippocampus volumes, CBF as well as the distribution of the standardised values of the AQ subscale scores and of the standardised composite positive schizotypy scores are provided in the Supplementary Material.

### Correlation between volume and CBF

In reference to previous work [72], we also analysed the correlation between structural and functional variation in the hippocampal sections, as well as voxel-wise within the whole hippocampus, independent of phenotype.

Sectional analyses were conducted by extracting individual volumes of three sections of hippocampi (e.g. head-, body and tail of hippocampus) and individual CBF values averaged over these volumes. A linear regression was then performed with age and gender as covariates to investigate the relationship between volume and resting CBF. We applied false discovery rate (FDR) correction for multiple comparisons across the three hippocampal sections of each hemisphere. In addition, we also repeated these correlation analyses controlling for positive schizotypy and each of the AQ subscales.

Voxel-wise analyses were conducted using the SPM software package (Statistical Parametric Mapping, Wellcome Centre for Human Neuroimaging, Institute of Neurology, London, UK) and the CAT12 toolbox (version 12.8, build r2137; Christian Gaser, Structural Brain Mapping Group, Jena University Hospital, Jena, Germany), running under Matlab 2019a. To that end, CBF images were co-registered to the volumetric images. For statistical analysis, we set up a full factorial model with a voxel-wise covariate and estimated the model using the TFCE (Threshold-Free Cluster Enhancement) toolbox.

## Results

### Hippocampal volumes

**Fig. 1:**
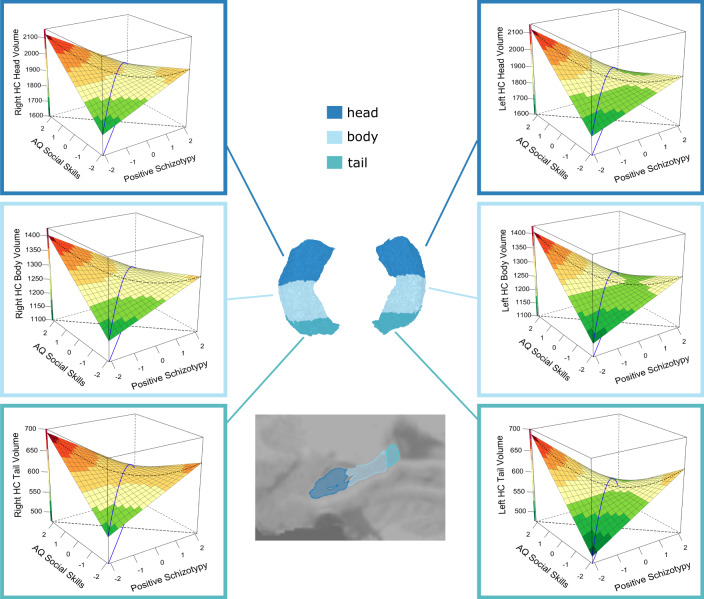
Response surface analysis (RSA) of the interactive association of positive schizotypy and AQ social skills with left and right head, body and tail hippocampal volumes (mm^3^). The blue line is the balance axis, and the black dotted line is the bias axis, where the balance axis represents equal expressions of autistic and positive schizotypy traits, and the bias axis (orthogonal to the balance axis) represents the relative and progressive dominance of autistic and/or positive schizotypy traits. Figures show a curvilinear relationship between these traits and hippocampal volumes, with volumes being larger along the bias axis and particularly in individuals with an AQ social skills-dominant trait profile. This was significant for the left head (β(se) = 38.26 (10.36), 95%CI= (17.96, 58.55), *p* < .001), left body (β(se) = 19.57 (8.34), 95%CI= (3.23, 35.91), *p* = 0.019), left tail (β(se) = 13.68 (4.69), 95%CI = (4.49, 22.87), *p* = 0.004), as well as for the right head (β(se) = 37.93 (12.06), 95%CI= (14.31,61.56), *p* = 0.002), right body (β(se) = 17.82 (6.64), 95%CI= (4.81, 30.84), *p* = 0.007), and right tail (β(se) = 11.27 (5.20), 95%CI = (1.08, 21.45), *p* = 0.030). Volumes were also smaller in individuals with relatively balanced levels of positive schizotypy and AQ social skills, and particularly in individuals with either low-low or high-high trait profiles, but this was significant only for the tail bilaterally (Left: β(se) = −20.37 (5.69), 95%CI = (−31.53, −9.22), *p* < .001; Right: β(se) = −11.97 (5.91), 95%CI = (−23.55, −0.38), *p* = .043). Surface colour-coding represents the volume of the hippocampus from low (green) to high (red).

**Fig. 2:**
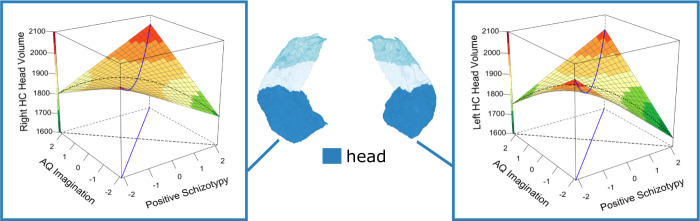
Response surface analysis (RSA) of the interactive association of positive schizotypy and AQ imagination with left and right head hippocampal volumes (mm^3^). The blue line is the balance axis, and the black dotted line is the bias axis, where the balance axis represents equal expressions of autistic and positive schizotypy traits, and the bias axis (orthogonal to the balance axis) represents the relative and progressive dominance of autistic and/or positive schizotypy traits. Figures show a curvilinear relationship between these traits and hippocampal volumes, with volumes being smaller, although nonsignificant (*p* > 0.05), along the bias axis and particularly in individuals with a positive schizotypy-dominant trait profile, and larger in individuals with relatively balanced levels of positive schizotypy and AQ imagination, and particularly in individuals with either low-low or high-high trait profiles (Left: β(se) = 34.93 (14.36), 95%CI = (6.79, 63.08), *p =* .015; Right: β(se) = 52.46 (15.46), 95%CI = (22.16, 82.75), *p* < .001). Surface colour-coding represents the volume of the hippocampus from low (green) to high (red).

The results of Pillai’s Trace of the MANCOVA for hippocampal volumes showed that only the positive schizotypy x social skills interaction (Pillai’s Trace Value = 0.060, F(6, 298) = 3.15, *p* = 0.005, ηp^2^ = 0.060), and the positive schizotypy x imagination interaction (Pillai’s Trace Value = 0.048, F(6, 298) = 2.51, *p* = 0.022, ηp^2^ = 0.048) were significant predictors. Beta estimates showed a significant negative association between the positive schizotypy x social skills interaction with both left and right whole hippocampal volumes and all subfields (see Table 1, Fig. 1). The legend of Fig. 1 reports the results of the RSA of these interactions. Conversely, beta estimates showed that the positive schizotypy x imagination interaction was positively associated with both left and right whole hippocampal volumes and specifically with the head subfield volume (see Table 2, Fig. 2). The legend of Fig. 2 reports the results of the RSA of these interactions.

**Table 1:** Beta estimates of the interactive association of positive schizotypy and social skills on total and subfield hippocampal volumes.

Region	B	SE	t_(df=303)_	p	Lower 95% CI	Upper 95% CI	Cohen’s d
Left Hippocampal tail	−20.84	5.28	3.95	0.000	−31.24	−10.45	0.45
Left Hippocampal body	−25.90	8.51	3.04	0.003	−42.65	−9.15	0.35
Left Hippocampal head	−34.38	12.96	2.65	0.008	−59.87	−8.89	0.30
Left Whole hippocampus	−81.13	22.24	3.65	0.000	−124.88	−37.37	0.42
Right Hippocampal tail	−17.87	5.313	3.37	0.001	−28.32	−7.42	0.39
Right Hippocampal body	−18.78	8.42	2.23	0.026	−35.35	−2.21	0.26
Right Hippocampal head	−32.01	13.01	2.46	0.014	−57.62	−6.41	0.28
Right Whole hippocampus	−68.66	22.42	3.06	0.002	−112.78	−24.54	0.35

*Β* parameter estimate; *SE* Standard Error, *t* value of t statistics; *df* Degrees of Freedom; *p* value of significance (significant if *p* < 0.05); *L* *CI* Lower bound of confidence interval; *U CI* Upper bound of confidence interval; *Cohen’s d* Effect size.

**Table 2:** Beta estimates of the interactive association of positive schizotypy and imagination on total and subfield hippocampal volumes.

Region	B	SE	t_(df=303)_	p	Lower 95% CI	Upper 95% CI	Cohen’s d
Left Hippocampal tail	7.41	4.31	1.72	0.087	−1.07	15.89	0.20
Left Hippocampal body	13.60	6.94	1.94	0.053	−0.25	37.88	0.22
Left Hippocampal head	38.38	10.57	3.63	0.000	17.59	59.18	0.42
Left Whole hippocampus	59.39	18.14	3.27	0.001	23.69	95.08	0.38
Right Hippocampal tail	7.31	4.33	1.69	0.092	−0.60	23.18	0.19
Right Hippocampal body	9.12	6.87	1.33	0.185	−4.39	22.64	0.15
Right Hippocampal head	28.09	10.61	2.65	0.009	7.20	48.97	0.30
Right Whole hippocampus	44.52	18.29	2.44	0.015	8.54	80.51	0.28

*Β* parameter estimate; *SE* Standard Error, *t* value of t statistics; *df* Degrees of Freedom; *p* value of significance (significant if *p* < 0.05); *L CI* Lower bound of confidence interval; *U CI* Upper bound of confidence interval; *Cohen’s d* Effect size.

### Hippocampal cerebral blood flow (CBF)

**Fig. 3:**
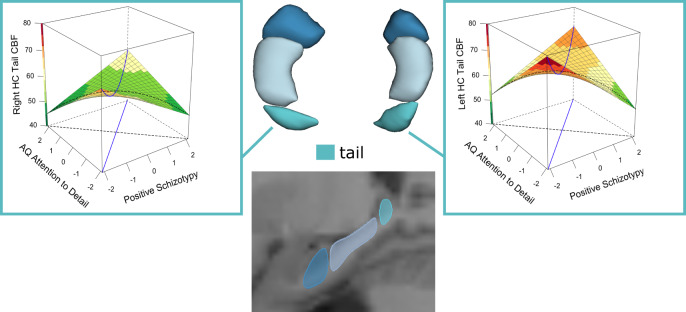
Response surface analysis (RSA) of the interactive association of positive schizotypy and AQ attention to detail with left and right tail hippocampal cerebral blood flow (CBF in ml/100 mg/min). The blue lines are the balance and bias axes, where the balance axis represents equal expressions of autistic and positive schizotypy traits, and the bias axis (orthogonal to the balance axis) represents the relative and progressive dominance of autistic and/or positive schizotypy traits. Figures show a curvilinear relationship between these traits and hippocampal volumes, with volumes being smaller along the bias axis and particularly in individuals with either positive schizotypy-dominant or an AQ attention to detail-dominant trait profile, and larger in individuals with relatively balanced levels of positive schizotypy and AQ attention to detail, and particularly in individuals with either low-low or high-high trait profiles. However, this curvilinearity was only significant for the association of the balance axis with CBF of the left hippocampal tail (Left: β(se) = 2.44 (0.81), 95%CI= (0.85, 4.03), *p =* 0.003; Right: β(se) = −3.24 (1.93), 95%CI = (−7.02, 0.55), *p* = .094). Surface colour-coding represents the CBF of the hippocampus from low (green) to high (red).

The results of Pillai’s Trace of the MANCOVA for CBF showed a significant effect only for increased focus of attention (AQ attention switching; Pillai’s Trace Value = 0.041, F(6, 326) = 2.33, *p* = 0.033, ηp^2^ = 0.041), and for the interaction of positive schizotypy x attention to detail (Pillai’s Trace Value = 0.043, F(6, 326) = 2.46, *p* = 0.024, ηp^2^ = 0.043). Beta estimates showed a positive, but a nonsignificant, association between increased focus of attention and right head hippocampal CBF (β(se) = 1.27(.72), t_(df=333)_ = 1.76, *p* = 0.079, Cohen’s d = 0.19). Beta estimates showed that the positive schizotypy x attention to detail interaction was positively and significantly associated with CBF of both the left (β(se) = 2.74(.99), t_(df=333)_ = 2.76, *p* = 0.006, Cohen’s d = 0.31) and right (β(se) = 2.11(1.01), t_(df=333)_ = 2.08, *p* = 0.038, Cohen’s d = 0.23) hippocampal tail (see Fig. 3). The legend of Fig. 3 reports the results of the RSA of these interactions.

### Correlation between volume and rCBF

**Fig. 4:**
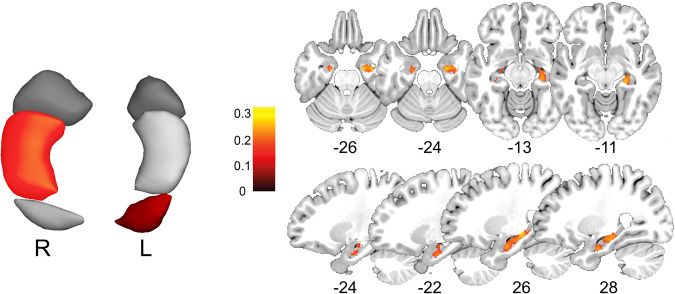
Correlation between volume and cerebral blood flow (independent of phenotype) in the six hippocampal sections (left). Results show significant positive correlations between volume and CBF in the left hippocampal tail (T(df = 295) = 2.268, p_uncorrected_ = 0.024) and the right hippocampal body (T(295) = 2.846, p_uncorrected_ = 0.005; p_FDR_ = 0.028) and in a voxel-wise analysis (right) within a mask containing both whole hippocampi. Colours indicate sections and voxels with significant correlations and correlation strength.

In the sectional analyses, we observed, as can be seen in Table 3, small but significant positive correlation between volume and CBF in the left hippocampal tail (T(df=295) = 2.268, p_uncorrected_ = 0.024) and the right hippocampal body (T(295) = 2.846, p_uncorrected_ = 0.005), as well as a trend for the right hippocampal head (T(295) = 1.953, p_uncorrected_ = 0.052). In the other sections, this association was not significant. However, only the right hippocampal body survived FDR correction (p_FDR_ = 0.028). This association remained significant after controlling for each of the AQ subscales and positive schizotypy (all p_s_ < 0.05). Significant results of the voxel-wise analysis (FWE-peak-level correction with *p* < 0.05) are visualised in Fig. 4.

**Table 3:** Correlation between volume and CBF of left and right subfield hippocampal volumes, controlling for age and gender.

Region	r	t_(df=295)_	P_uncorrected_	P_FDR_
Left Hippocampal tail	0.13	2.27	0.024	0.072
Left Hippocampal body	0.05	0.86	0.393	0.393
Left Hippocampal head	0.09	1.54	0.149	17.59
Right Hippocampal tail	0.10	1.70	0.089	0.134
Right Hippocampal body	0.16	2.85	0.005	0.028
Right Hippocampal head	0.11	1.95	0.052	0.103

## Discussion

In this study, we address the problem of overlap and interaction of psychiatric spectrum phenotypes using a refined dimensional model of autistic vs. schizotypal traits in a cohort with subclinical trait expression. This novel approach allows us to expand a previously developed and evaluated psychometric model [7, 26, 73] towards establishing an alternative framework for the study of co-morbidities in psychiatric disorders based on biological data. Our findings challenge the notion that brain volume loss is a general feature of schizophrenia spectrum phenotypes [74], and contrary to previously held assertions [75, 76], demonstrating that decreased volumes can occur independent of the effect of antipsychotic drugs. This is also in line with recent studies of hippocampal volume and perfusion in schizotypy [19, 42], as well as clinical high-risk and developing psychosis [39, 51]. These changes might indicate neurodevelopmental failure or a state marker of conversion to psychosis proneness [77]. As such, our findings might suggest different aetiologies or pathways leading to brain structural or functional changes. Our approach might thus be useful in facilitating clinical trial enrichment and stratification, as well as precision diagnostics, expanding designs to consider subclinical phenotypes and phenotype overlaps [78, 79].

Our findings provide at least three major novel implications for precision psychiatry approaches: first, they demonstrate that hippocampal volumes and regional blood flow are not only sensitive to subclinical expression of either schizotypy and autistic traits, but that these phenotypes interact, resulting in more complex nonlinear effects. In the case of psychosis-ASD overlap, such effects have been hypothesised from phenotype and cognitive studies [31, 80, 81] and clinical studies of social functioning [82], but hardly for biological markers [18]. Among our main findings is the observation of a pervasive negative interactive association of positive schizotypy and AQ social skills with whole and subfield hippocampal volumes in both hemispheres. Response surface analysis revealed that a shift from a positive schizotypy-dominant to AQ social skills-dominant trait profile followed a curvilinear pattern, with volumes being larger in individuals with an AQ social skills-dominant trait profile. Intriguingly, volumes were smaller in individuals with relatively balanced levels of positive schizotypy and AQ social skills, and particularly in individuals with either low-low or high-high trait profiles. Hence, the combination of two (rather than one isolated) spectrum markers more adequately predicted regional volume. This finding is important as it demonstrates that analysing multiple disease spectra in conjunction is a more powerful and adequate approach to mapping psychopathology on brain circuits. This also helps explain seemingly paradoxical findings, such as the combination of high positive schizotypy and high AQ-social skills resulting in preserved hippocampal volumes. These findings may suggest compensation across mechanisms associated with ASD and SSD. We reason that this compensatory mechanism is plausible, given previous studies reporting that ASD was associated with increased volume [83, 84] and SSD with reduced volume [20, 85, 86]. Collectively, the co-occurrence of ASD and SSD may yield effects that are protective of normative brain structures, which mirrors previously observed effects of ASD and SSD on brain and social functioning [17, 18]. Main effects only models are thus likely to yield incomplete information as to the association between brain-disease specific phenotypes. Moreover, the specificity of the association of positive schizotypy and AQ social skills with hippocampal volumes highlights the ever-increasing significant role of the hippocampus for social processes [44, 87], core features of both ASD and SSD. Volume of a brain structure is, however, determined by multiple factors, including not only cell number or size, but possibly also regional cerebral blood volume and flow. In interpreting our findings, it is thus important to acknowledge that it remains unclear how different molecular or cellular level changes or pathways might result in an overall volume effect. For example, in schizophrenia, volume and neuron numbers are reduced in the hippocampus [86], but the underlying post mortem studies cannot infer on the timing of these changes. Hence, these processes might only commence after disease onset or might in part be prevalent in subjects at higher risk, such as those with high (positive) schizotypy. In contrast, even in the absence of cellular-level (micro)structural changes, the potential overlap in GABAergic activity in parvalbumin-positive neurons to hippocampal dysfunction in schizophrenia and autism [88, 89] might be relevant to a common pathway. Although our study cannot infer on which of these mechanisms might relate to the macroscopic variation in hippocampal volume (and blood flow), our findings make the case that in the study of a disease spectrum, phenotype data from other complementary clinical spectra can yield complementary information to better understand effects that might result in masking or unmasking of neural-level processes.

An important aspect and point of relevance for clinical studies is that our findings demonstrate (on the phenotype facet level) an equifinality of outcomes when combining multiple phenotypes. In other terms, reduction of hippocampal volume (or subfields) might emerge as a result of multiple combinations of risk factors or psychopathologies combined. This would add an additional perspective on recent large-scale patient studies and meta-analyses, which have demonstrated shared regional or pathway pathologies across multiple psychiatric disorders [1, 90, 91] – yet the lack of regional specificity might have been due to lack of facet-level phenotyping as well as their interaction. Hence, “common” effects in transdiagnostic studies might actually be the result of “obscured/unmeasured” single facet effects.

Second, our subfield analysis approach also indicates that these interactions are not uniformly observed across the hippocampus, but that different subregions of the hippocampus are differentially sensitive to these effects, with an additional dissociation of structural vs. functional effects. These are reflected in the two other major findings, i.e., the positive interactive association of positive schizotypy and AQ imagination scores on the total hippocampus and the hippocampal head subfield volumes, as well as the positive interaction for rCBF with positive schizotypy and AQ attention to detail phenotypes, where effects were prominent in the left and right hippocampal tail only. This indicates at least two dissociations, i.e., by hippocampal region as well as for structure (volumes) vs. function (rCBF). Indeed, recent association studies for schizotypy have failed to identify correlations with total hippocampal volume per se [92], while subfield analyses do report effects on particular subregions related to subclinical psychotic-like features [20]. Moreover, studies have found increased [83, 84], decreased [93] and no association [94, 95] of hippocampal volumes with ASD. One potential source for this inconsistency in results is variation of unmeasured co-occurring subclinical expressions.

Third, and finally, our findings provide first empirical evidence for a fully dimensional approach taking into account the interaction of disease spectra, showing how co-occurring phenotypes might interact to affect functional and structural outcome. This has implications for future analysis embracing precision psychiatry. Most studies of putative biomarkers in psychiatry use case-control studies, such as brain volume comparisons of schizophrenia patients vs. healthy controls [96] or multiple diagnostic groups vs. healthy controls [1]. However, such categorical comparisons typically consider clinical “supra-threshold” expressions of psychiatric phenotypes, vastly neglecting variance within both cases and controls, and only consider disease-related phenotypes as a whole (rather than particular facets of the phenotype). Even with the development of recent novel meta-analytic approaches to co-morbidity mapping [97], they fail to consider how comorbid conditions interact to influence outcome or the ubiquitous overlap with other disease spectra, including the subclinical expression or psychopathology other than the ones under study.

**Fig. 5:**
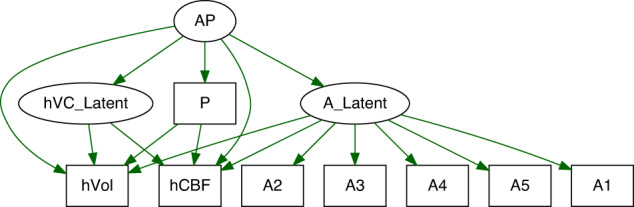
Candidate Structural Equation Model (SEM) examining the association of autistic traits (A_Latent), positive schizotypy (P), and their interaction (AP) on each of the hippocampal volume (hVol) and hippocampal CBF (hCBF) measures, and on the factor representing their relationship (hVC_Latent). A priori sample size power analysis for this model, with 3 latent and 8 observed variables, the minimum sample size required to detect a small effect size is 1,258 (f^2^ = 0.1, 1-β = 0.80, α = 0.05), and 1,624 at 0.90 power. Figure was simulated using the *lavaan* package in R.

Our study used both structural and functional imaging markers for the hippocampus and its subregions. Several mechanistic models of cerebral blood flow or cerebral blood volume argue that hyperactivity at the onset of psychosis might drive grey matter loss in the hippocampus [72, 88]. Particularly, dysfunction of parvalbumin positive inhibitory interneurons was linked to increased cerebral blood volume in mouse models of psychosis. In fact, multiple human studies detected hyperactivity in the ventral hippocampus of subjects with early psychosis [37–41]. In addition, reduced hippocampal grey matter volume and altered shape have been reported from both neuroimaging and post-mortem studies of psychosis [72, 77, 85, 86, 98], with a predominance for the left anterior hippocampus. The timing of these group differences suggested that hippocampal hyperactivity precedes symptom onset and hippocampal volume decrease [88]. Also, glutamate levels in the hippocampus correlated inversely with hippocampal volumes in unmedicated first-episode psychosis [85]. Finally, a recent mouse model of ErbB4 mutants demonstrated that dysfunctional inhibitory interneurons may drive increased CBF and glutamine levels in the ventral hippocampus, while glutamate and GABA levels remained unchanged [43]. While timing and extent of structural and perfusion changes in the hippocampus may differ between subjects with psychosis and individuals with schizotypal traits, our study suggests that this link is also relevant in autism albeit with a different pattern. For example, reports of increased hippocampus perfusion in autism are less consistent [48, 99, 100]. Yet, changes in excitation and inhibition are capable of shifting the balance between factors that define the metabolic cost of a brain region as well as the formation of axonal connections, and ultimately volume [48, 99]. To the extent that rCBF/hippocampal volume variations are a reflection of excitatory and inhibitory processes within the hippocampus, we invoke findings from a magnetic resonance spectroscopy study to substantiate this possibility, in which we showed that autistic and positive schizotypal traits interactively predicted the balance between excitatory (glutamate) and inhibitory (GABA+) neurotransmitter concentrations in the superior temporal cortex—a region involved in social language and functioning [101] —and that excitation/inhibition imbalance is associated with shared psychosocial deficits across the ASD and SSD spectra [102]. Similarly, higher CBF was shown to correlate with lower N-acetylaspartate (NAA) levels in frontal white matter of adults with autism [48]. Therefore, it is plausible that association between rCBF and hippocampal volume are modulated by the interaction of autistic traits and schizotypy. Since our data did not permit examining the interaction between autistic traits and positive schizotypy with the interaction between rCBF and hippocampal volume, it would be important for future research to explore these complex associations with properly powered structural equation models (see Fig. 5 for details). Our analyses do, however, allow the interpretation that volume and rCBF are coupled in the hippocampus, albeit with considerable regional variation (see Fig. 4), and that this seems to be a basic feature in healthy subjects, independent of variations in positive schizotypy or autistic-like phenotypes.

Recently, Parkes, Satterthwaite and Bassett (2020) have argued for biomarker studies to consider transdiagnostic research designs, dimensional models of psychopathology, as well as modelling throughout development [79]. Such approaches would, however, require not only multiple patient cohorts, but also a more fine-grained psychopathology assessment at the facet level. Many case-control studies fail on these requirements – not only because they mostly consider only one patient group, but also because they usually phenotype only for one disease spectrum (e.g., psychotic symptoms in a schizophrenia cohort or mood in affective disorder studies). Our approach, while applied to subclinical phenotypes, adds multi-dimensional phenotyping to this strategy, whereby multiple disease spectra are assessed and facets (e.g., positive schizotypy or social skills) rather than global psychopathological markers (e.g., psychosis severity) are considered for outcomes. While this approach could be translated to established clinical cohorts, many of these seem to lack phenotyping for multiple disease spectra. In addition, transdiagnostic phenotyping might require sensitive instruments that can detect variations across diagnostic boundaries, such as impulsivity or aggression [14], as well as validation across clinical spectra.

The use of this novel approach carries some limitations. First, while the validity of both schizotypal traits and autistic-like traits as part of respective disease continua is based on a wealth of studies and data (e.g. [32, 35, 103]), it is not undisputed [104], and for autism, for example, a more narrow definition of the phenotype has recently been advocated [104]. Our approach makes use of an extended phenotype conceptualisation of both ASD and schizophrenia spectrum disorders (SSD). Yet, the strong presence of substantial comorbidity in most psychiatric axis I disorders clearly calls for spectrum models, given that subthreshold symptoms or traits are often present in any given established psychiatric diagnostic category [105]. Second, our analysis so far is limited to structural data and rCBF of the hippocampus, which was chosen in a hypothesis-driven approach, given its relevance for most psychiatric disorders as well as both cognitive and social functions [87, 106, 107]. Task-based fMRI might be useful to study particular cognitive functions related to the hippocampus (or its subregions) and the spectrum overlap. Finally, our approach focused on positive schizotypy, given its established diametric relationship with certain autistic-like features as well as clinical studies of this symptom spectrum in psychosis.

In conclusion, our findings provide first empirical evidence for the interactive effects of two disease spectra (schizophrenia/psychosis vs. ASD) on structure and function across multiple hippocampal subfields. This provides a blueprint to studying convergence of psychiatric disease spectra (rather than categories), resolving requirements suggesting that that informing etiological and phenotypic overlaps between two diseases would require the utilisation of a dual-diagnosis cohort compared with two control groups, each singly diagnosed with one or other [108]. By using dimensional measures that cut across diagnostic boundaries, our approach makes tangible the development of a multidimensional model for understanding the relationship between two disease spectra, and to uncover how different disease combinations might affect an outcome within the individual.

### Supplementary information

Supplementary Material

## Data Availability

Data are available upon reasonable request and pending local and national data protection and ethics regulations.

## References

[1] Opel N, Goltermann J, Hermesdorf M, Berger K, Baune BT, Dannlowski U (2020). Cross-disorder analysis of brain structural abnormalities in six major psychiatric disorders: a secondary analysis of mega- and meta-analytical findings from the ENIGMA Consortium. Biol Psychiatry.

[2] Baker JT, Dillon DG, Patrick LM, Roffman JL, Brady RO, Pizzagalli DA (2019). Functional connectomics of affective and psychotic pathology. Proc Natl Acad Sci USA.

[3] Kushki A, Anagnostou E, Hammill C, Duez P, Brian J, Iaboni A (2019). Examining overlap and homogeneity in ASD, ADHD, and OCD: a data-driven, diagnosis-agnostic approach. Transl Psychiatry.

[4] Padberg F, Bulubas L, Mizutani-Tiebel Y, Burkhardt G, Kranz GS, Koutsouleris N (2021). The intervention, the patient and the illness - Personalizing non-invasive brain stimulation in psychiatry. Exp Neurol.

[5] Thompson PM, Jahanshad N, Ching CRK, Salminen LE, Thomopoulos SI, Bright J (2020). ENIGMA and global neuroscience: A decade of large-scale studies of the brain in health and disease across more than 40 countries. Transl Psychiatry.

[6] Brainstorm Consortium, Anttila V, Bulik-Sullivan B, Finucane HK, Walters RK, Bras J et al. Analysis of shared heritability in common disorders of the brain. *Science* 2018;360. 10.1126/science.aap8757 PMC6097237 29930110

[7] Chisholm K, Lin A, Abu-Akel A, Wood SJ (2015). The association between autism and schizophrenia spectrum disorders: A review of eight alternate models of co-occurrence. Neurosci Biobehav Rev.

[8] Williams LM (2016). Precision psychiatry: a neural circuit taxonomy for depression and anxiety. Lancet Psychiatry.

[9] Moreau CA, Raznahan A, Bellec P, Chakravarty M, Thompson PM, Jacquemont S (2021). Dissecting autism and schizophrenia through neuroimaging genomics. Brain.

[10] Goodkind M, Eickhoff SB, Oathes DJ, Jiang Y, Chang A, Jones-Hagata LB (2015). Identification of a common neurobiological substrate for mental illness. JAMA Psychiatry.

[11] McTeague LM, Huemer J, Carreon DM, Jiang Y, Eickhoff SB, Etkin A (2017). Identification of common neural circuit disruptions in cognitive control across psychiatric disorders. Am J Psychiatry.

[12] McTeague LM, Rosenberg BM, Lopez JW, Carreon DM, Huemer J, Jiang Y (2020). Identification of common neural circuit disruptions in emotional processing across psychiatric disorders. Am J Psychiatry.

[13] Walton E, Hibar DP, van Erp TGM, Potkin SG, Roiz-Santianez R, Crespo-Facorro B (2018). Prefrontal cortical thinning links to negative symptoms in schizophrenia via the ENIGMA consortium. Psychol Med.

[14] Wong TY, Radua J, Pomarol-Clotet E, Salvador R, Albajes-Eizagirre A, Solanes A (2020). An overlapping pattern of cerebral cortical thinning is associated with both positive symptoms and aggression in schizophrenia via the ENIGMA consortium. Psychol Med.

[15] Nenadic I, Sauer H, Gaser C (2010). Distinct pattern of brain structural deficits in subsyndromes of schizophrenia delineated by psychopathology. Neuroimage.

[16] Zhang T, Koutsouleris N, Meisenzahl E, Davatzikos C (2015). Heterogeneity of structural brain changes in subtypes of schizophrenia revealed using magnetic resonance imaging pattern analysis. Schizophr Bull.

[17] Abu-Akel A, Wood SJ, Upthegrove R, Chisholm K, Lin A, Hansen PC (2022). Psychosocial functioning in the balance between autism and psychosis: evidence from three populations. Mol Psychiatry.

[18] Abu-Akel A, Apperly IA, Wood SJ, Hansen PC (2017). Autism and psychosis expressions diametrically modulate the right temporoparietal junction. Soc Neurosci.

[19] Sahakyan L, Meller T, Evermann U, Schmitt S, Pfarr JK, Sommer J (2021). Anterior vs posterior hippocampal subfields in an extended psychosis phenotype of multidimensional schizotypy in a nonclinical sample. Schizophr Bull.

[20] Evermann U, Gaser C, Meller T, Pfarr JK, Grezellschak S, Nenadic I (2021). Nonclinical psychotic-like experiences and schizotypy dimensions: Associations with hippocampal subfield and amygdala volumes. Hum Brain Mapp.

[21] Clark LA, Cuthbert B, Lewis-Fernandez R, Narrow WE, Reed GM (2017). Three Approaches to Understanding and Classifying Mental Disorder: ICD-11, DSM-5, and the National Institute of Mental Health’s Research Domain Criteria (RDoC). Psychol Sci Public Interest.

[22] Insel T, Cuthbert B, Garvey M, Heinssen R, Pine DS, Quinn K (2010). Research domain criteria (RDoC): toward a new classification framework for research on mental disorders. Am J Psychiatry.

[23] Krueger RF, Kotov R, Watson D, Forbes MK, Eaton NR, Ruggero CJ (2018). Progress in achieving quantitative classification of psychopathology. World Psychiatry.

[24] Kastner A, Begemann M, Michel TM, Everts S, Stepniak B, Bach C (2015). Autism beyond diagnostic categories: characterization of autistic phenotypes in schizophrenia. BMC Psychiatry.

[25] Searles Quick VB, Davis JM, Olincy A, Sikela JM (2015). DUF1220 copy number is associated with schizophrenia risk and severity: implications for understanding autism and schizophrenia as related diseases. Transl Psychiatry.

[26] Nenadic I, Meller T, Evermann U, Schmitt S, Pfarr JK, Abu-Akel A (2021). Subclinical schizotypal vs. autistic traits show overlapping and diametrically opposed facets in a non-clinical population. Schizophr Res.

[27] Zhou HY, Yang HX, Gong JB, Cheung EFC, Gooding DC, Park S (2019). Revisiting the overlap between autistic and schizotypal traits in the non-clinical population using meta-analysis and network analysis. Schizophr Res.

[28] Nair A, Jolliffe M, Lograsso YSS, Bearden CE (2020). A review of default mode network connectivity and its association with social cognition in adolescents with autism spectrum disorder and early-onset psychosis. Front Psychiatry.

[29] Solmi M, Radua J, Olivola M, Croce E, Soardo L, Salazar de Pablo G et al. Age at onset of mental disorders worldwide: large-scale meta-analysis of 192 epidemiological studies. *Mol Psychiatry* 2021. 10.1038/s41380-021-01161-7 PMC8960395 34079068

[30] Schwarz K, Moessnang C, Schweiger JI, Baumeister S, Plichta MM, Brandeis D (2020). Transdiagnostic prediction of affective, cognitive, and social function through brain reward anticipation in schizophrenia, bipolar disorder, major depression, and autism spectrum diagnoses. Schizophr Bull.

[31] Fernandes JM, Cajao R, Lopes R, Jeronimo R, Barahona-Correa JB (2018). Social cognition in schizophrenia and autism spectrum disorders: a systematic review and meta-analysis of direct comparisons. Front Psychiatry.

[32] Massrali A, Brunel H, Hannon E, Wong C, Baron-Cohen S, i P-MEG (2019). Integrated genetic and methylomic analyses identify shared biology between autism and autistic traits. Mol Autism.

[33] Ruzich E, Allison C, Smith P, Watson P, Auyeung B, Ring H (2015). Measuring autistic traits in the general population: a systematic review of the Autism-Spectrum Quotient (AQ) in a nonclinical population sample of 6,900 typical adult males and females. Mol Autism.

[34] Blanchette CA, Amirova J, Bohbot VD, West GL (2019). Autistic traits in neurotypical individuals are associated with increased landmark use during navigation. Psych J.

[35] Nelson MT, Seal ML, Pantelis C, Phillips LJ (2013). Evidence of a dimensional relationship between schizotypy and schizophrenia: a systematic review. Neurosci Biobehav Rev.

[36] Tonini E, Quide Y, Kaur M, Whitford TJ, Green MJ (2021). Structural and functional neural correlates of schizotypy: A systematic review. Psychol Bull.

[37] Allen P, Azis M, Modinos G, Bossong MG, Bonoldi I, Samson C (2018). Increased resting hippocampal and basal ganglia perfusion in people at ultra high risk for psychosis: replication in a second cohort. Schizophr Bull.

[38] Allen P, Chaddock CA, Egerton A, Howes OD, Bonoldi I, Zelaya F (2016). Resting hyperperfusion of the hippocampus, midbrain, and basal ganglia in people at high risk for psychosis. Am J Psychiatry.

[39] Schobel SA, Chaudhury NH, Khan UA, Paniagua B, Styner MA, Asllani I (2013). Imaging patients with psychosis and a mouse model establishes a spreading pattern of hippocampal dysfunction and implicates glutamate as a driver. Neuron.

[40] Schobel SA, Lewandowski NM, Corcoran CM, Moore H, Brown T, Malaspina D (2009). Differential targeting of the CA1 subfield of the hippocampal formation by schizophrenia and related psychotic disorders. Arch Gen Psychiatry.

[41] McHugo M, Talati P, Armstrong K, Vandekar SN, Blackford JU, Woodward ND (2019). Hyperactivity and reduced activation of anterior hippocampus in early psychosis. Am J Psychiatry.

[42] Modinos G, Egerton A, McMullen K, McLaughlin A, Kumari V, Barker GJ (2018). Increased resting perfusion of the hippocampus in high positive schizotypy: A pseudocontinuous arterial spin labeling study. Hum Brain Mapp.

[43] Kiemes A, Serrano Navacerrada ME, Kim E, Randall K, Simmons C, Rojo Gonzalez L (2023). Erbb4 deletion from inhibitory interneurons causes psychosis-relevant neuroimaging phenotypes. Schizophr Bull.

[44] Banker SM, Gu X, Schiller D, Foss-Feig JH (2021). Hippocampal contributions to social and cognitive deficits in autism spectrum disorder. Trends Neurosci.

[45] Carlisi CO, Norman L, Murphy CM, Christakou A, Chantiluke K, Giampietro V (2017). Shared and disorder-specific neurocomputational mechanisms of decision-making in autism spectrum disorder and obsessive-compulsive disorder. Cereb Cortex.

[46] DeRamus TP, Kana RK (2015). Anatomical likelihood estimation meta-analysis of grey and white matter anomalies in autism spectrum disorders. Neuroimage Clin.

[47] Tang S, Liu X, Ran Q, Nie L, Wu L, Pan Z (2022). Application of three-dimensional pseudocontinuous arterial spin labeling perfusion imaging in the brains of children with autism. Front Neurol.

[48] Peterson BS, Zargarian A, Peterson JB, Goh S, Sawardekar S, Williams SCR (2019). Hyperperfusion of frontal white and subcortical gray matter in autism spectrum disorder. Biol Psychiatry.

[49] Li Y, Shen M, Stockton ME, Zhao X (2019). Hippocampal deficits in neurodevelopmental disorders. Neurobiol Learn Mem.

[50] Genon S, Bernhardt BC, La Joie R, Amunts K, Eickhoff SB (2021). The many dimensions of human hippocampal organization and (dys)function. Trends Neurosci.

[51] Provenzano FA, Guo J, Wall MM, Feng X, Sigmon HC, Brucato G (2020). Hippocampal pathology in clinical high-risk patients and the onset of schizophrenia. Biol Psychiatry.

[52] First MB, Gibbon M The Structured Clinical Interview for DSM-IV Axis I Disorders (SCID-I) and the Structured Clinical Interview for DSM-IV Axis II Disorders (SCID-II). Comprehensive handbook of psychological assessment, Vol. 2: *Personality assessment*. John Wiley & Sons Inc: Hoboken, NJ, US, 2004, pp 134-43.

[53] Wittchen H-U, Wunderlich U, Gruschwitz S, Zaudig M SKID-I. *Strukturiertes Klinisches Interview für DSM-IV*. Hogrefe: Göttingen, 1997.

[54] Lehrl S, Triebig G, Fischer B (1995). Multiple choice vocabulary test MWT as a valid and short test to estimate premorbid intelligence. Acta Neurol Scand.

[55] World Medical Association. (2013). World Medical Association Declaration of Helsinki: ethical principles for medical research involving human subjects. JAMA.

[56] Raine A (1991). The SPQ: a scale for the assessment of schizotypal personality based on DSM-III-R criteria. Schizophr Bull.

[57] Mason O, Claridge G (2006). The Oxford-Liverpool Inventory of Feelings and Experiences (O-LIFE): further description and extended norms. Schizophr Res.

[58] Mason O, Claridge G, Jackson M (1995). New scales for the assessment of schizotypy. Pers Indiv Diff.

[59] Kwapil TR, Gross GM, Burgin CJ, Raulin ML, Silvia PJ, Barrantes-Vidal N (2018). Validity of the Multidimensional Schizotypy Scale: Associations with schizotypal traits and normal personality. Personal Disord.

[60] Kwapil TR, Gross GM, Silvia PJ, Raulin ML, Barrantes-Vidal N (2018). Development and psychometric properties of the Multidimensional Schizotypy Scale: A new measure for assessing positive, negative, and disorganized schizotypy. Schizophr Res.

[61] Baron-Cohen S, Wheelwright S, Skinner R, Martin J, Clubley E (2001). The autism-spectrum quotient (AQ): evidence from Asperger syndrome/high-functioning autism, males and females, scientists and mathematicians. J Autism Dev Disord.

[62] English MCW, Gignac GE, Visser TAW, Whitehouse AJO, Maybery MT (2020). A comprehensive psychometric analysis of autism-spectrum quotient factor models using two large samples: Model recommendations and the influence of divergent traits on total-scale scores. Autism Res.

[63] Happe F, Ronald A (2008). The ‘fractionable autism triad’: a review of evidence from behavioural, genetic, cognitive and neural research. Neuropsychol Rev.

[64] Beck AT, Ward CH, Mendelson M, Mock J, Erbaugh J (1961). An inventory for measuring depression. Arch Gen Psychiatry.

[65] Hautzinger M (1991). [The Beck Depression Inventory in clinical practice]. Nervenarzt.

[66] Fischl B (2012). FreeSurfer. Neuroimage.

[67] Fischl B, Salat DH, Busa E, Albert M, Dieterich M, Haselgrove C (2002). Whole brain segmentation: automated labeling of neuroanatomical structures in the human brain. Neuron.

[68] Iglesias JE, Augustinack JC, Nguyen K, Player CM, Player A, Wright M (2015). A computational atlas of the hippocampal formation using ex vivo, ultra-high resolution MRI: Application to adaptive segmentation of in vivo MRI. Neuroimage.

[69] Eggert LD, Sommer J, Jansen A, Kircher T, Konrad C (2012). Accuracy and reliability of automated gray matter segmentation pathways on real and simulated structural magnetic resonance images of the human brain. PLoS One.

[70] Wang J, Aguirre GK, Kimberg DY, Roc AC, Li L, Detre JA (2003). Arterial spin labeling perfusion fMRI with very low task frequency. Magn Reson Med.

[71] Lenth RV (2009). Response-Surface Methods in R. Using rsm J Stat Softw.

[72] Lieberman JA, Girgis RR, Brucato G, Moore H, Provenzano F, Kegeles L (2018). Hippocampal dysfunction in the pathophysiology of schizophrenia: a selective review and hypothesis for early detection and intervention. Mol Psychiatry.

[73] Abu-Akel A, Clark J, Perry A, Wood SJ, Forty L, Craddock N (2017). Autistic and schizotypal traits and global functioning in bipolar I disorder. J Affect Disord.

[74] Wannan CMJ, Cropley VL, Chakravarty MM, Bousman C, Ganella EP, Bruggemann JM (2019). Evidence for Network-Based Cortical Thickness Reductions in Schizophrenia. Am J Psychiatry.

[75] Ansell BR, Dwyer DB, Wood SJ, Bora E, Brewer WJ, Proffitt TM (2015). Divergent effects of first-generation and second-generation antipsychotics on cortical thickness in first-episode psychosis. Psychol Med.

[76] Vita A, De Peri L, Deste G, Barlati S, Sacchetti E (2015). The effect of antipsychotic treatment on cortical gray matter changes in schizophrenia: does the class matter? A meta-analysis and meta-regression of longitudinal magnetic resonance imaging studies. Biol Psychiatry.

[77] Roeske MJ, McHugo M, Vandekar S, Blackford JU, Woodward ND, Heckers S (2021). Incomplete hippocampal inversion in schizophrenia: prevalence, severity, and impact on hippocampal structure. Mol Psychiatry.

[78] Chand GB, Dwyer DB, Erus G, Sotiras A, Varol E, Srinivasan D (2020). Two distinct neuroanatomical subtypes of schizophrenia revealed using machine learning. Brain.

[79] Parkes L, Satterthwaite TD, Bassett DS (2020). Towards precise resting-state fMRI biomarkers in psychiatry: synthesizing developments in transdiagnostic research, dimensional models of psychopathology, and normative neurodevelopment. Curr Opin Neurobiol.

[80] Abu-Akel A, Apperly I, Spaniol MM, Geng JJ, Mevorach C (2018). Diametric effects of autism tendencies and psychosis proneness on attention control irrespective of task demands. Sci Rep.

[81] Crespi B, Badcock C (2008). Psychosis and autism as diametrical disorders of the social brain. Behav Brain Sci.

[82] Isvoranu AM, Ziermans T, Schirmbeck F, Borsboom D, Geurts HM, de Haan L (2022). Autistic symptoms and social functioning in psychosis: a network approach. Schizophr Bull.

[83] Groen W, Teluij M, Buitelaar J, Tendolkar I (2010). Amygdala and hippocampus enlargement during adolescence in autism. J Am Acad Child Adolesc Psychiatry.

[84] Murphy CM, Deeley Q, Daly EM, Ecker C, O’Brien FM, Hallahan B (2012). Anatomy and aging of the amygdala and hippocampus in autism spectrum disorder: an in vivo magnetic resonance imaging study of Asperger syndrome. Autism Res.

[85] Kraguljac NV, White DM, Reid MA, Lahti AC (2013). Increased hippocampal glutamate and volumetric deficits in unmedicated patients with schizophrenia. JAMA Psychiatry.

[86] Roeske MJ, Konradi C, Heckers S, Lewis AS (2021). Hippocampal volume and hippocampal neuron density, number and size in schizophrenia: a systematic review and meta-analysis of postmortem studies. Mol Psychiatry.

[87] Tavares RM, Mendelsohn A, Grossman Y, Williams CH, Shapiro M, Trope Y (2015). A Map for Social Navigation in the Human Brain. Neuron.

[88] Heckers S, Konradi C (2015). GABAergic mechanisms of hippocampal hyperactivity in schizophrenia. Schizophr Res.

[89] Taylor SF, Grove TB, Ellingrod VL, Tso IF (2019). The Fragile Brain: Stress Vulnerability, Negative Affect and GABAergic Neurocircuits in Psychosis. Schizophr Bull.

[90] Bipolar D, Major Depressive D, Obsessive-Compulsive D, Schizophrenia EWG, Writing Committee for the Attention-Deficit/Hyperactivity D, Autism Spectrum D (2021). Virtual Histology of Cortical Thickness and Shared Neurobiology in 6 Psychiatric Disorders. JAMA Psychiatry.

[91] Koshiyama D, Fukunaga M, Okada N, Morita K, Nemoto K, Usui K (2020). White matter microstructural alterations across four major psychiatric disorders: mega-analysis study in 2937 individuals. Mol Psychiatry.

[92] Kirschner M, Hodzic-Santor B, Antoniades M, Nenadic I, Kircher T, Krug A et al. Cortical and subcortical neuroanatomical signatures of schizotypy in 3004 individuals assessed in a worldwide ENIGMA study. *Mol Psychiatry* 2021. 10.1038/s41380-021-01359-9 PMC9054674 34707236

[93] Eilam-Stock T, Wu T, Spagna A, Egan LJ, Fan J (2016). Neuroanatomical Alterations in High-Functioning Adults with Autism Spectrum Disorder. Front Neurosci.

[94] Aylward EH, Minshew NJ, Goldstein G, Honeycutt NA, Augustine AM, Yates KO (1999). MRI volumes of amygdala and hippocampus in non-mentally retarded autistic adolescents and adults. Neurology.

[95] Piven J, Bailey J, Ranson BJ, Arndt S (1998). No difference in hippocampus volume detected on magnetic resonance imaging in autistic individuals. J Autism Dev Disord.

[96] van Erp TG, Hibar DP, Rasmussen JM, Glahn DC, Pearlson GD, Andreassen OA (2016). Subcortical brain volume abnormalities in 2028 individuals with schizophrenia and 2540 healthy controls via the ENIGMA consortium. Mol Psychiatry.

[97] Fortea L, Albajes-Eizagirre A, Yao YW, Soler E, Verdolini N, Hauson AO (2021). Focusing on Comorbidity-A Novel Meta-Analytic Approach and Protocol to Disentangle the Specific Neuroanatomy of Co-occurring Mental Disorders. Front Psychiatry.

[98] Gutman BA, van Erp TGM, Alpert K, Ching CRK, Isaev D, Ragothaman A (2022). A meta-analysis of deep brain structural shape and asymmetry abnormalities in 2,833 individuals with schizophrenia compared with 3,929 healthy volunteers via the ENIGMA Consortium. Hum Brain Mapp.

[99] Jann K, Hernandez LM, Beck-Pancer D, McCarron R, Smith RX, Dapretto M (2015). Altered resting perfusion and functional connectivity of default mode network in youth with autism spectrum disorder. Brain Behav.

[100] Knudsen LV, Sheldrick AJ, Vafaee MS, Michel TM (2023). Diversifying autism neuroimaging research: An arterial spin labeling review. Autism.

[101] Ford TC, Abu-Akel A, Crewther DP (2019). The association of excitation and inhibition signaling with the relative symptom expression of autism and psychosis-proneness: Implications for psychopharmacology. Prog Neuropsychopharmacol Biol Psychiatry.

[102] Ford TC, Crewther DP, Abu-Akel A (2020). Psychosocial deficits across autism and schizotypal spectra are interactively modulated by excitatory and inhibitory neurotransmission. Autism.

[103] Abu-Akel A, Allison C, Baron-Cohen S, Heinke D (2019). The distribution of autistic traits across the autism spectrum: evidence for discontinuous dimensional subpopulations underlying the autism continuum. Mol Autism.

[104] Mottron L, Bzdok D (2020). Autism spectrum heterogeneity: fact or artifact?. Mol Psychiatry.

[105] Lewinsohn PM, Shankman SA, Gau JM, Klein DN (2004). The prevalence and co-morbidity of subthreshold psychiatric conditions. Psychol Med.

[106] Buckner RL (2010). The role of the hippocampus in prediction and imagination. Annu Rev Psychol.

[107] Zeidman P, Maguire EA (2016). Anterior hippocampus: the anatomy of perception, imagination and episodic memory. Nat Rev Neurosci.

[108] Larson FV, Wagner AP, Jones PB, Tantam D, Lai MC, Baron-Cohen S (2017). Psychosis in autism: comparison of the features of both conditions in a dually affected cohort. Br J Psychiatry.

