# Supplementary material for: Modelling the overlap and divergence of autistic and schizotypal traits on hippocampal subfield volumes and regional cerebral blood flow
Source: Mol Psychiatry. 2023 Oct 27;29(1):74–84. doi: 10.1038/s41380-023-02302-w (PMC11078729; doi:10.1038/s41380-023-02302-w)

Supplementary Material

1. Distribution of raw values for left and right tail, body, head, and whole hippocampus volumes


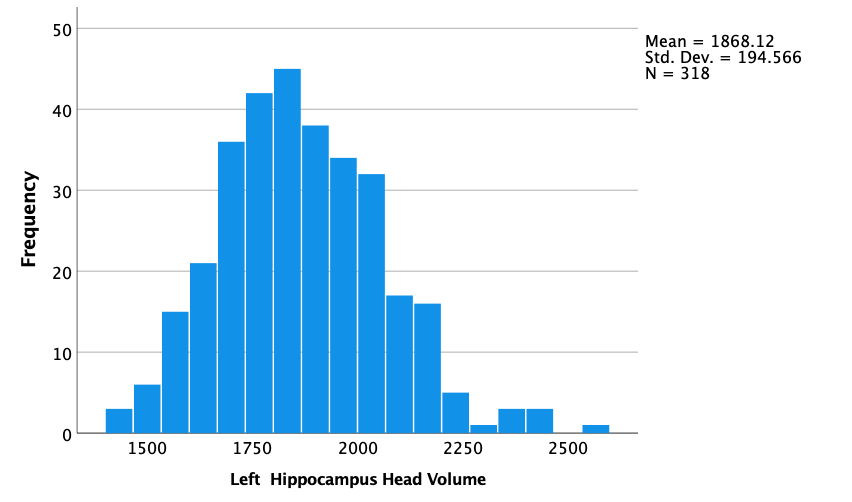

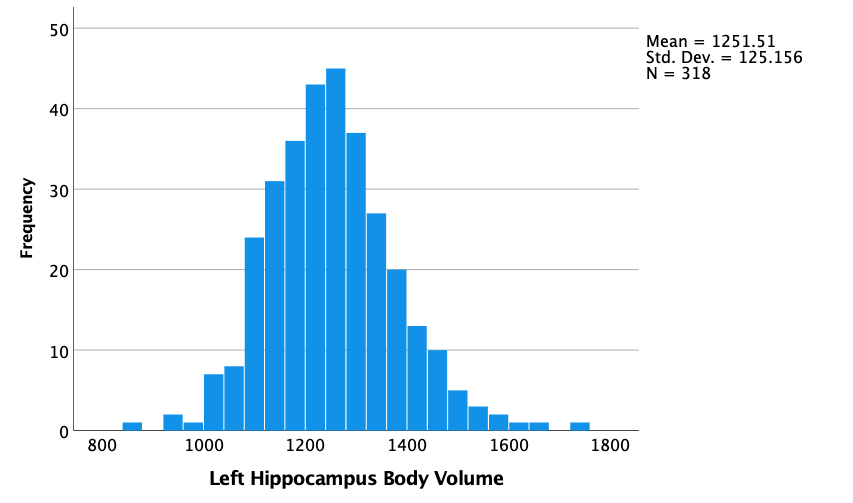


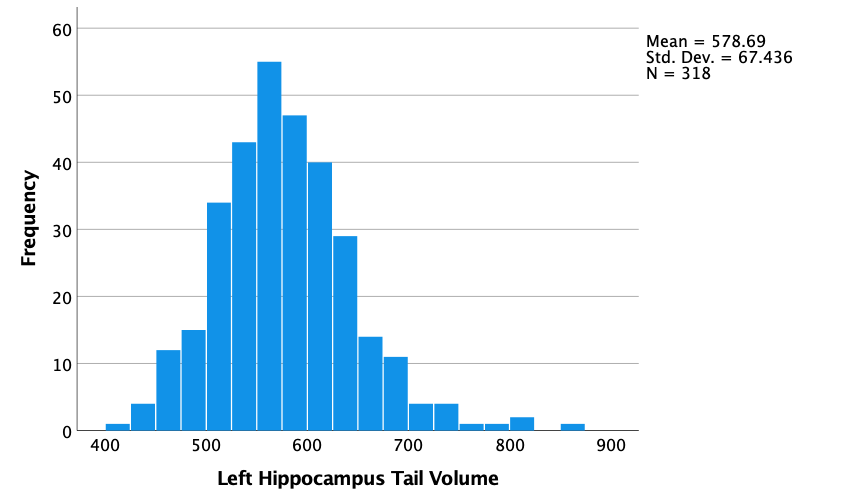

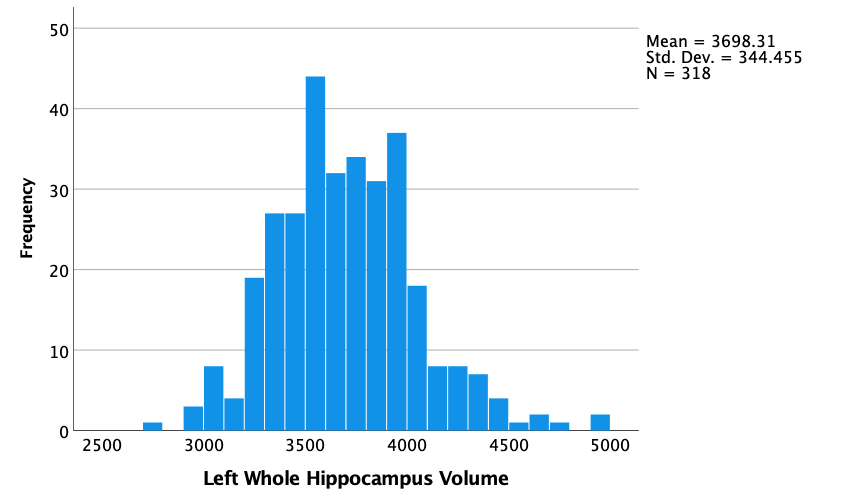


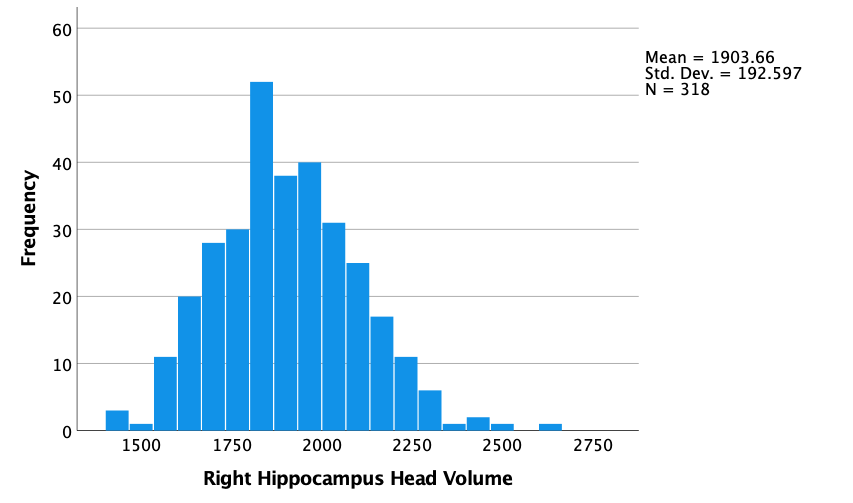

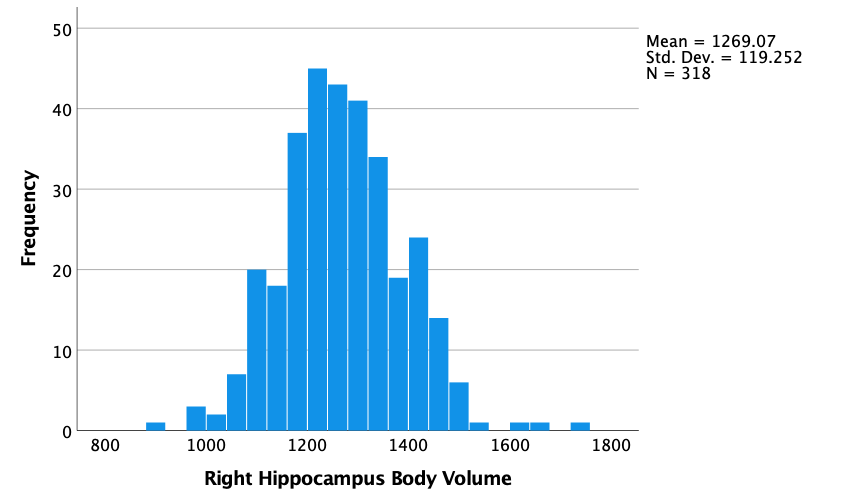


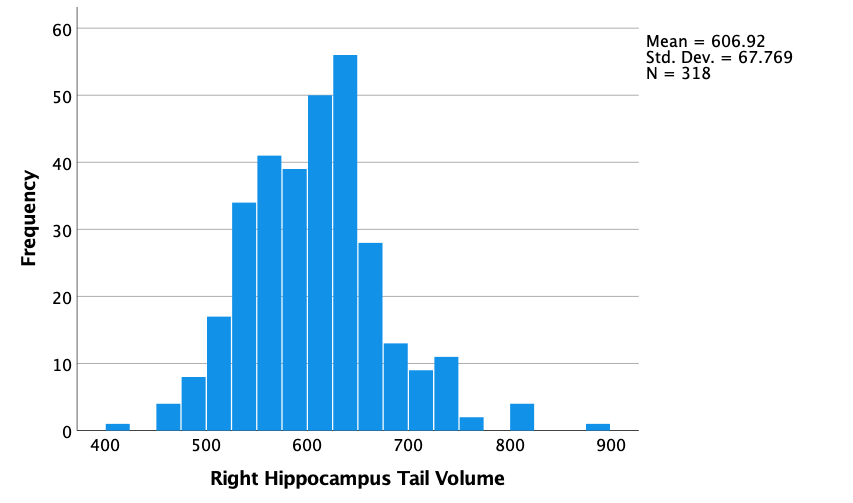

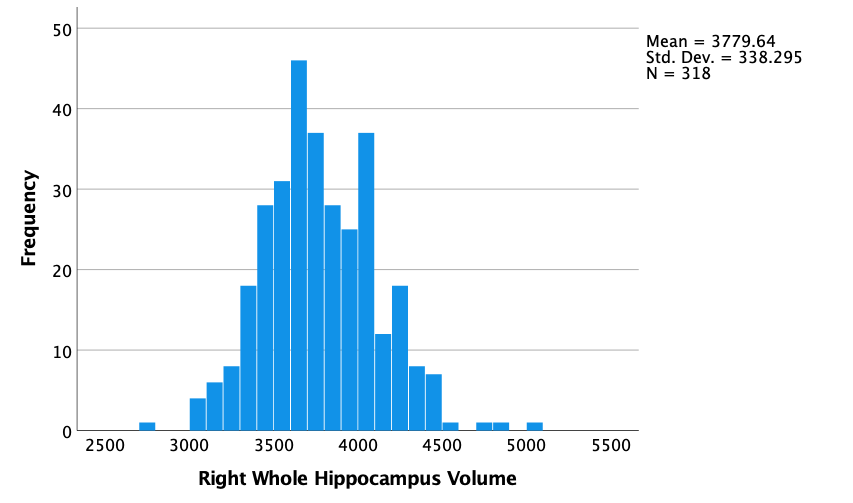


1. Distribution of standardized AQ subscale scores of the hippocampus volume sample (N=318)


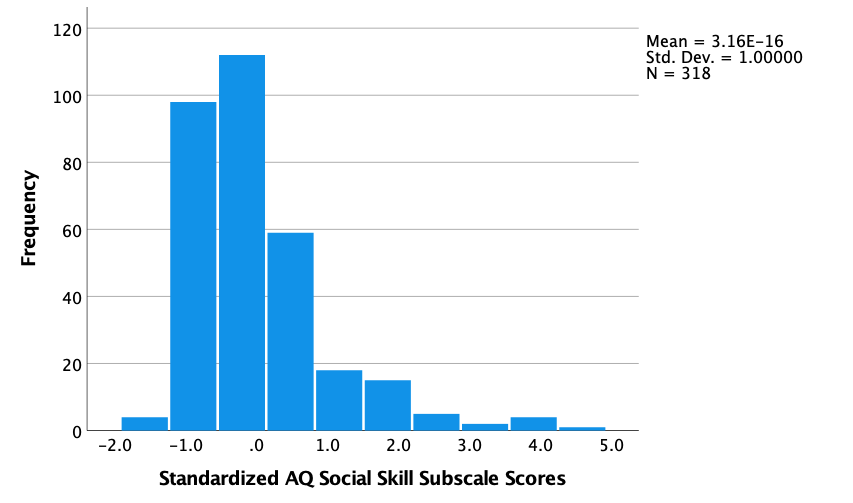

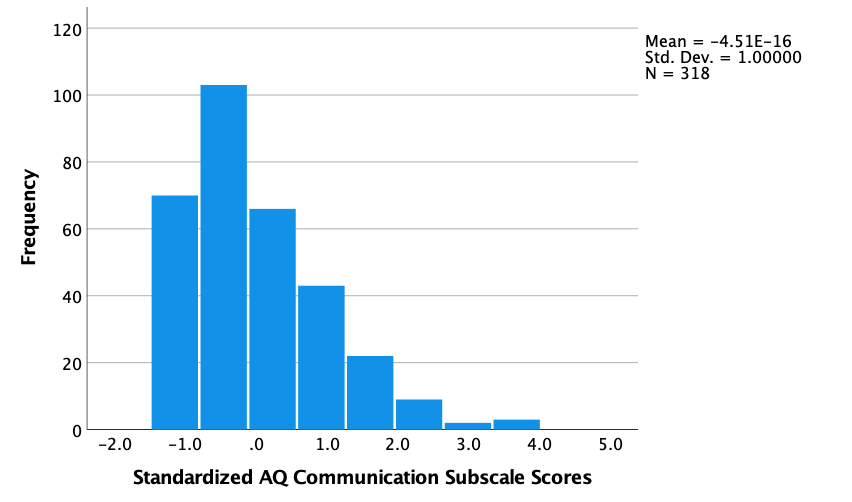

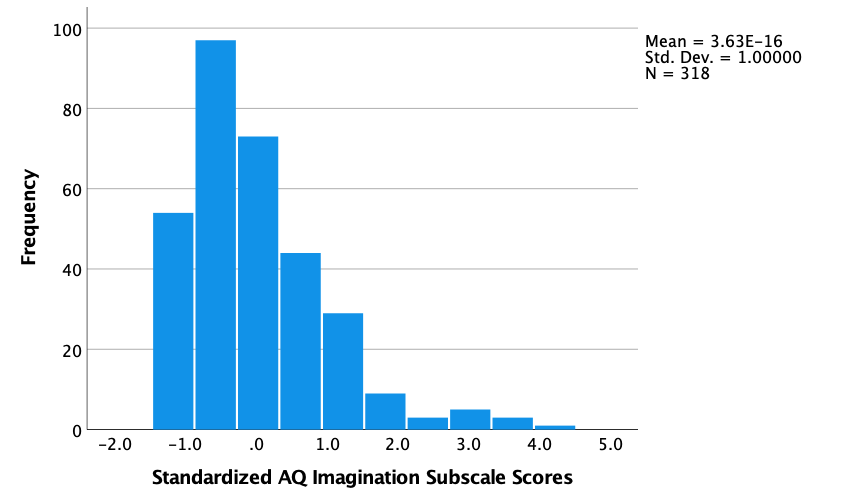

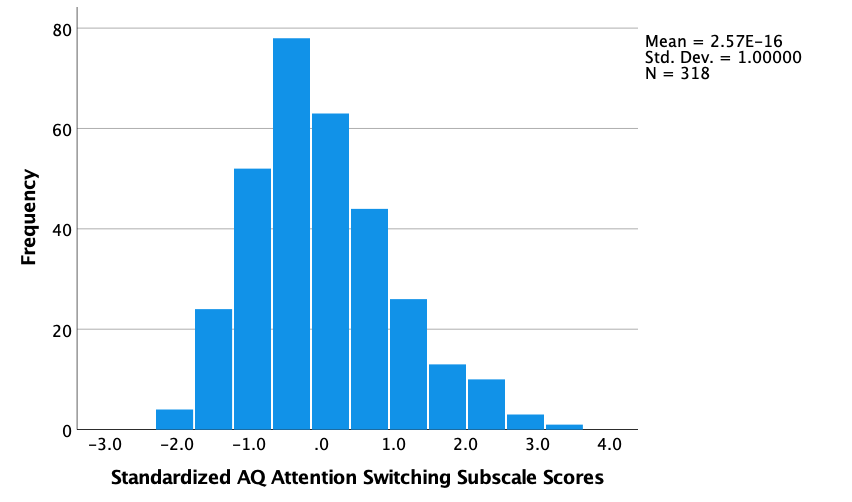

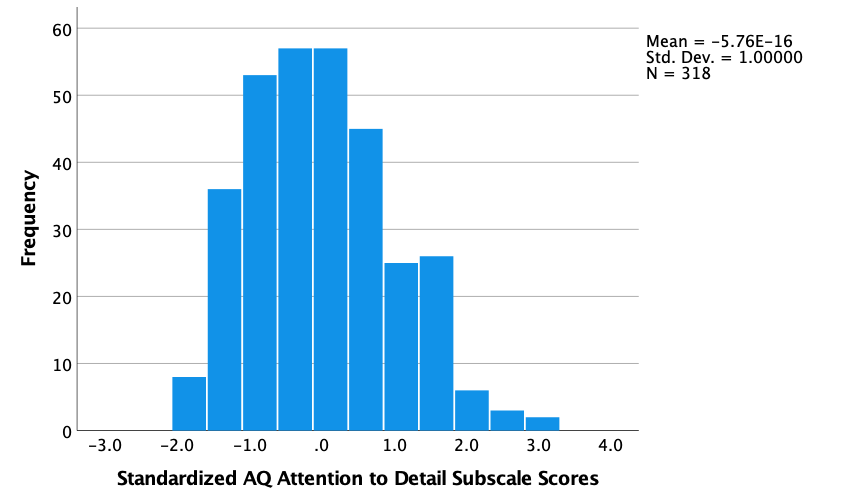


1. Distribution of the standardized composite score of positive schizotypy of the hippocampus volume sample (N=318)


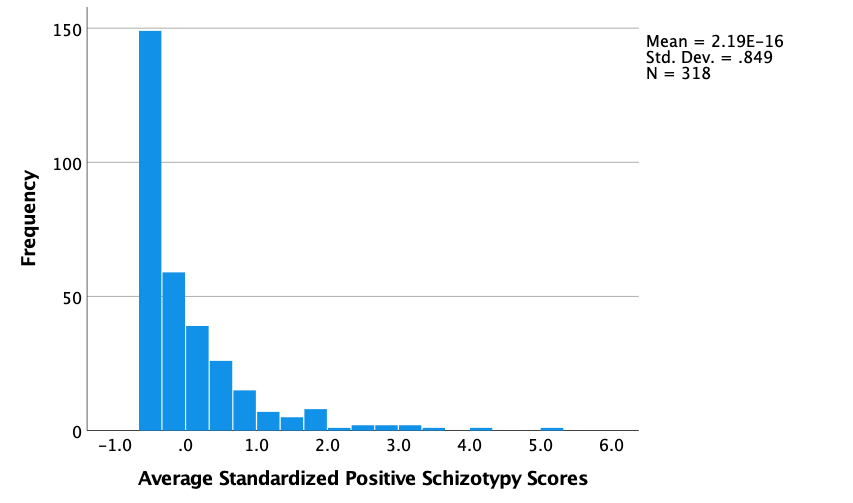


1. Distribution of raw values for left and right whole, head, boy and tail hippocampus cerebral blood flow (N=346)


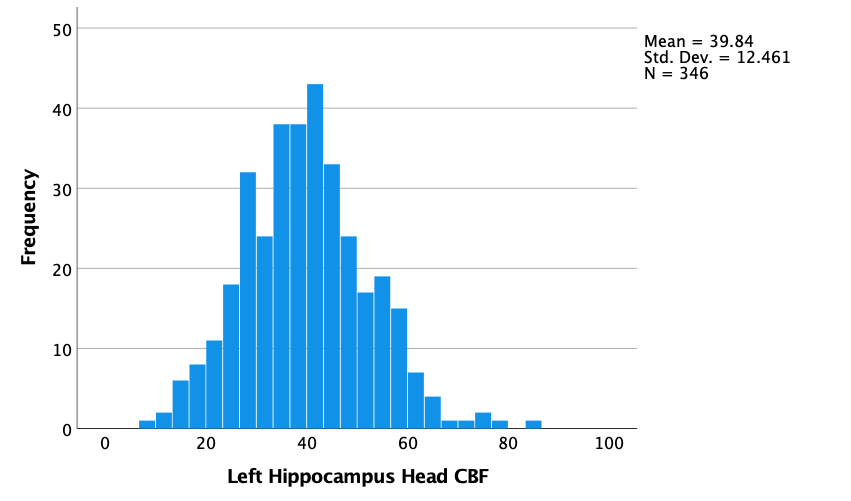

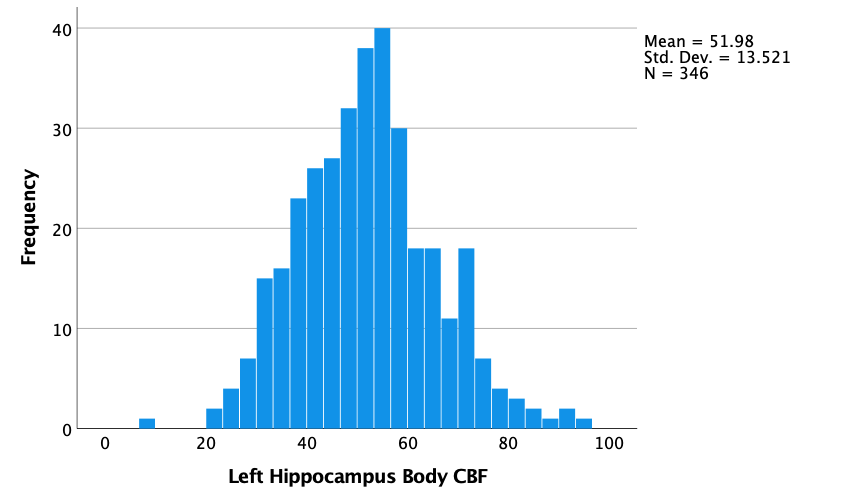


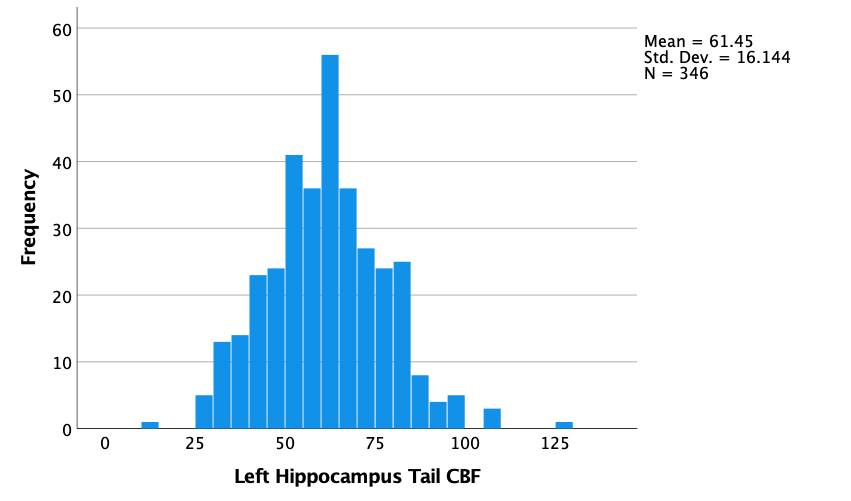

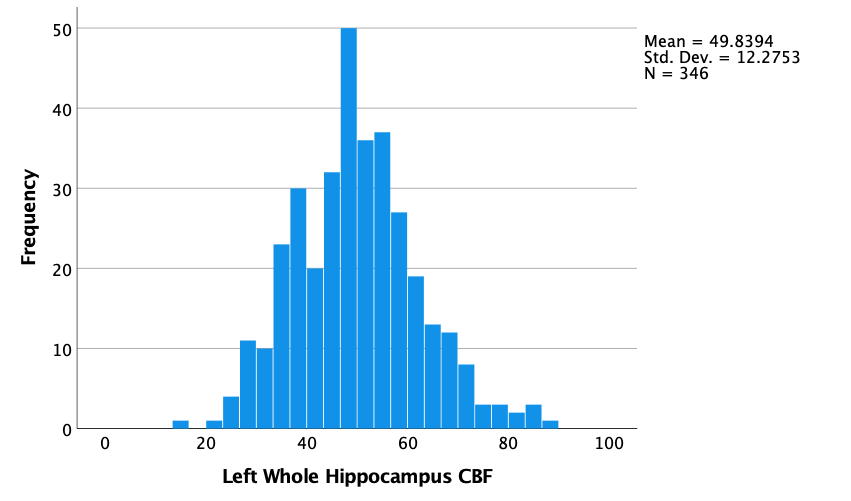


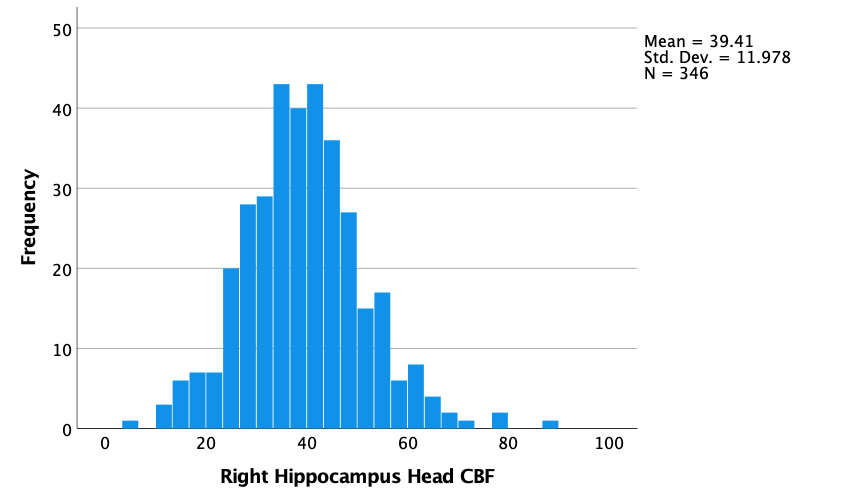

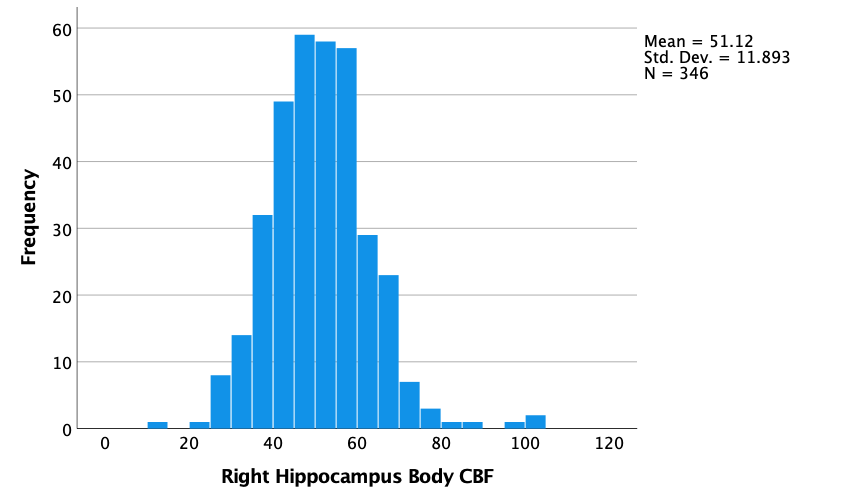


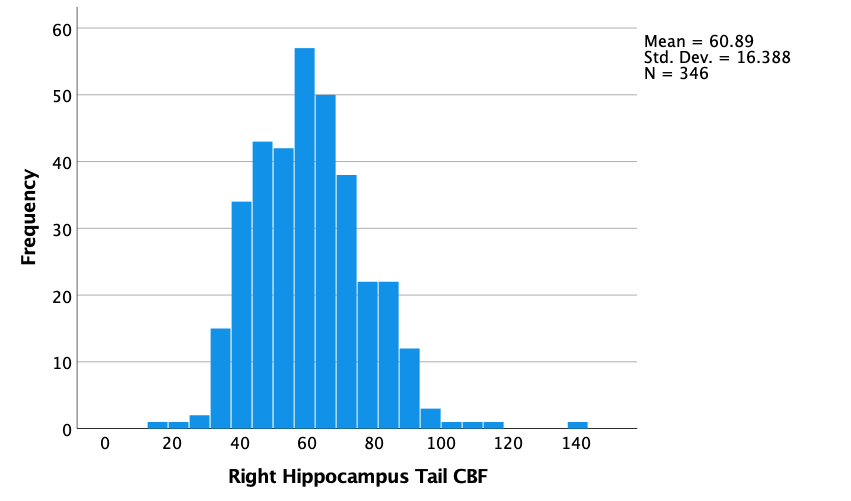

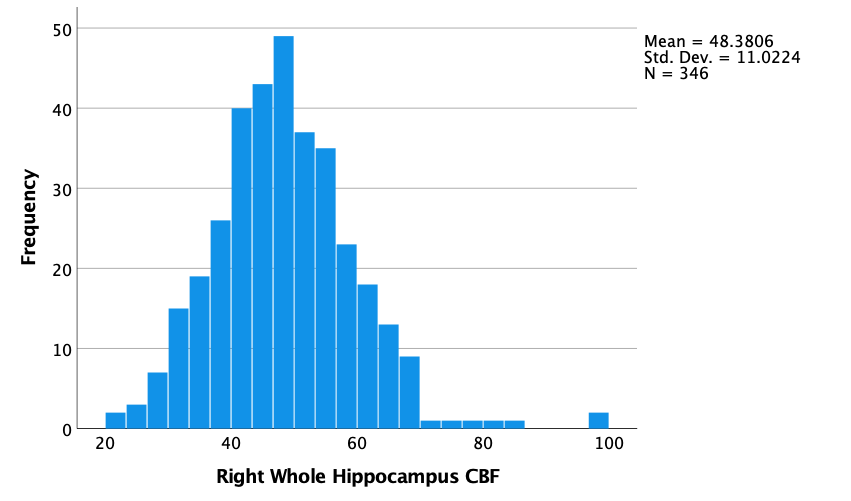


1. Distribution of standardized AQ subscale scores of the hippocampus CBF sample (N=346)


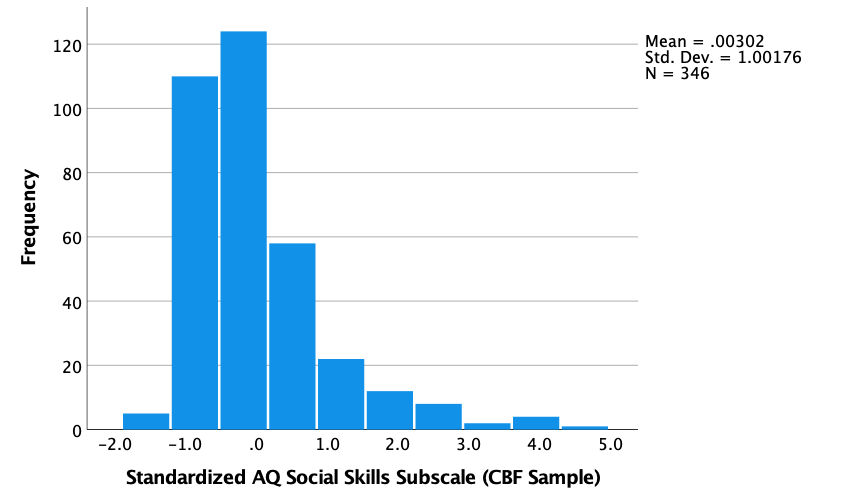

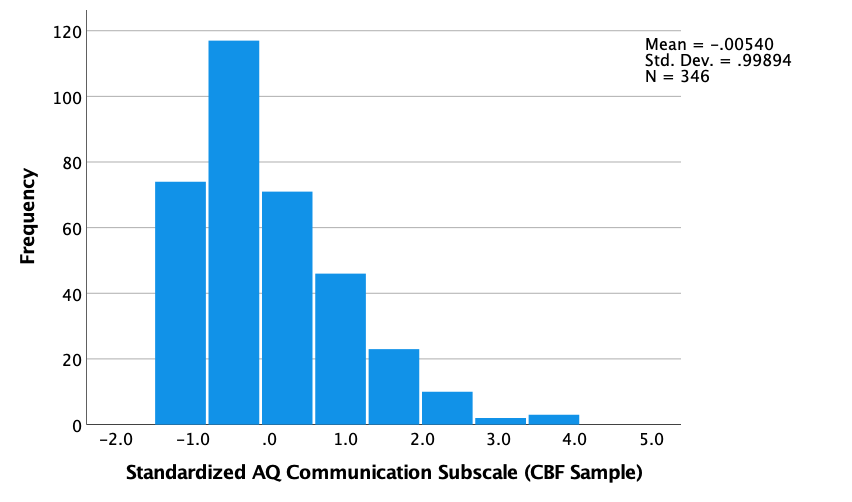


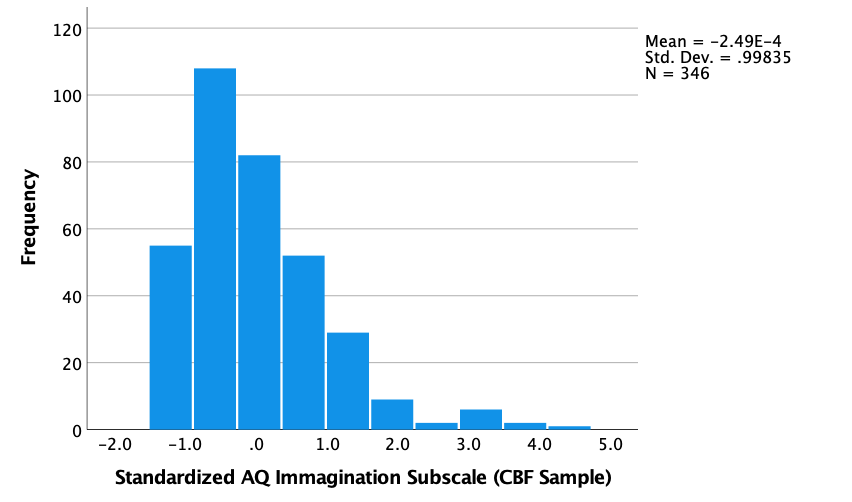

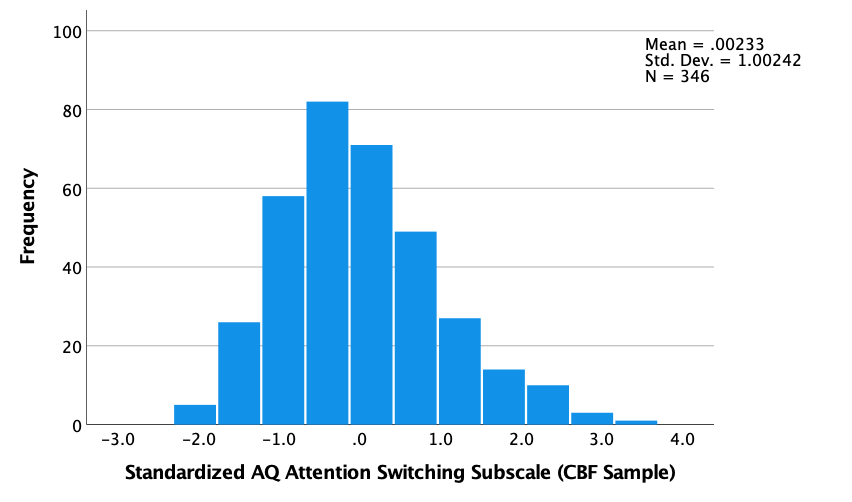

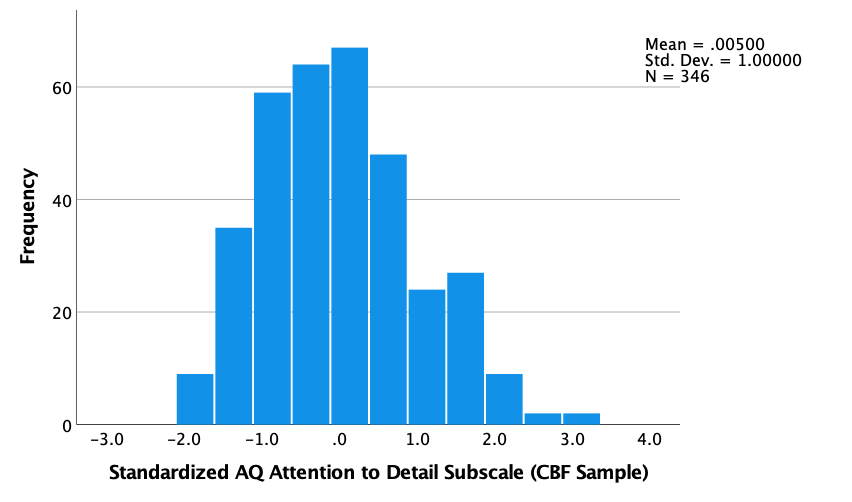


1. Distribution of the standardized composite score of positive schizotypy of the hippocampus CBF sample (N=346)


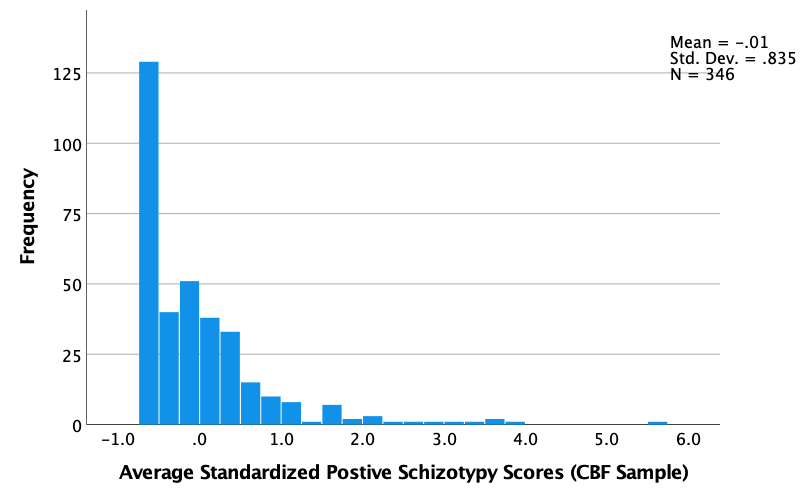

Supplement: Supplementary file 1 — Supplementary Material [file 41380_2023_2302_MOESM1_ESM.docx]
